# Histo-epidemiological aspects of gynecological and breast cancers at the University Teaching Hospital of Yaoundé

**DOI:** 10.11604/pamj.2019.33.130.18874

**Published:** 2019-06-21

**Authors:** Armand Duclaire Djimeli Kemo, Blaise Nkegoum, Charlette Nangue, Félicité Djuikwo, Landry Beyala Bita’a, Pierre Marie Tebeu

**Affiliations:** 1 Higher Institute of Health Sciences, Université des Montagnes, Bangangté, Cameroon; 2 Department of Pathological Anatomy, University Teaching Hospital of Yaoundé, Yaoundé, Cameroon; 3 Department of Parasitology, Université des Montagnes, Bangangté, Cameroon; 4 Meilleur Accès aux soins de Santé (M.A. SANTE), Yaoundé, Cameroon; 5 Gynecology-Obstetrics Service, University Teaching Hospital of Yaoundé, Yaoundé, Cameroon

**Keywords:** Gynecological cancers, epidemiology, histopathology, Yaoundé, Cameroon

## Abstract

**Introduction:**

Cancer is a public health problem that affect women more than men. The aim of the study was to describe the epidemiological and histopathological features of gynecological malignancies in the city of Yaoundé, Cameroon.

**Methods:**

This was a descriptive cross-sectional study of histologically proven gynecological cancers over a 10-year period (2008-2017) in the Gynecology and Pathological Anatomy Departments of the University Teaching Hospital of Yaoundé.

**Results:**

A total of 682 cancers were identified among which, 342 gynecological cancers, for an overall frequency of 50.1% and an annual frequency of 34.2 cases on average. There was a trend suggesting an increase annual frequency over time. The cervix was the most frequent location with 182 cases (53.2%); followed by breast with 96 cases (28.1%); endometrium with 33 cases (9.7%) and ovaries 15 cases (4.4%). These patients were on average 51.9±13.7 years old, mostly housewives (56.8%), married (60.4%), multiparous (61.3%) and referred (62.6%). Histopathologically, cervical cancer was predominantly squamous cell carcinoma (86.8%), invasive (80.9%) and well differentiated (45.5%). For breast cancers, the majority were ductal carcinomas (78.1%), invasive (92%), and histological grade SBR II (50.6%). The most common histopathological types of endometrial and ovarian cancer were adenocarcinoma (72.2%) and serous cystadenocarcinoma (46.7%), respectively.

**Conclusion:**

Gynecological cancers are common. Screening is expected to increase at 30 years for cervical cancer and start at age 40 with mammography for breast cancer.

## Introduction

Cancer is a group of serious diseases, fatal in the absence of effective treatment, characterized by a rapid and uncontrolled proliferation of abnormal cells that can affect any part of the body. When it touches the breast, the ovary or an organ of the genital tract, it is called gynecological cancer. Breast cancer is the most common cancer in the world with 1.7 million new cases, including 522,000 deaths each year [1]. Cervical cancer ranks second among women's cancers worldwide with 528,000 new cases and 266,000 deaths in 2012. Gynecological cancers account for 41.3% of all cancers in Africa [2]. They are responsible for 56.2% of cancer deaths among women in Cameroon [3]. Several studies have been conducted on gynecological cancers in Africa in general and in Cameroon in particular; however, few of them have been carried out at the University Teaching Hospital of Yaoundé, yet it is a reference hospital involved in the diagnosis and treatment of cancers. Therefore, we proposed to conduct this study, to help strengthen the prevention of these cancers.

## Methods

**Figure 1:**
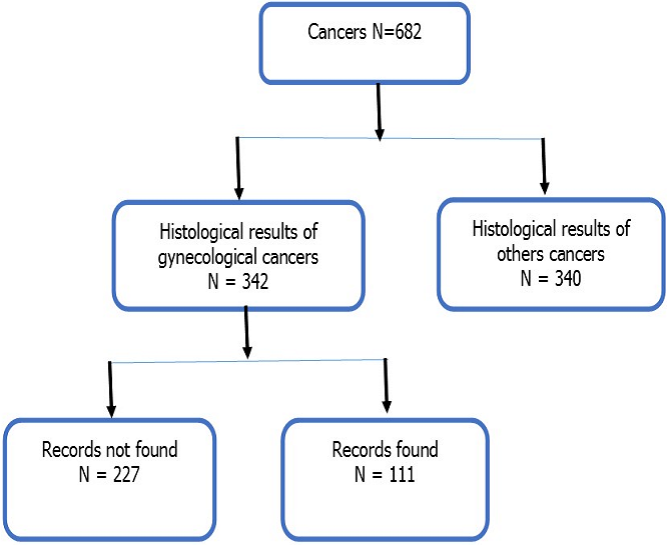
File selection profile

**Figure 2:**
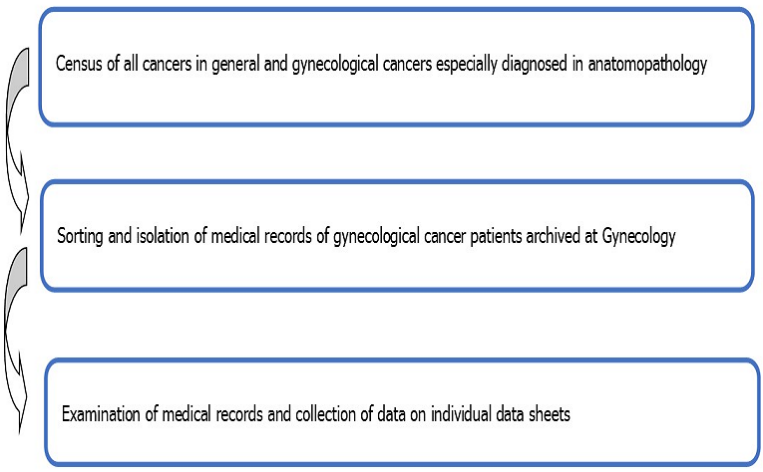
Data collection procedure

Institutional ethic committee of Université des Montagnes approved this study; authorization N°2018/002/UdM/PR/CIE and research authorization from the General Director of University Teaching Hospital of Yaoundé, was obtained for the realization of this study. This was a descriptive cross-sectional study carried out in the Gynecology-Obstetrics and Pathological Anatomy Departments of the University Teaching Hospital of Yaoundé over a period of 10 years from January 1, 2008 to December 31, 2017. The study material consisted of Gynecology-Obstetrics and Pathological Anatomy registers; medical records and individual data sheets. Sampling was non-probabilistic, consecutive and exhaustive of all histopathological findings and or medical records confirming the diagnosis of in situ or invasive cancer of the breast, ovary or any organ of the genital tract. The case selection profile is presented in Figure 1. The data collection procedure is presented in Figure 2. The data were analyzed using the Epi info 7.1.3.3 software; the variables studied were frequency, age, marital status, occupation, religion, place of residence, cancer site, histological type, invasive character or not, the degree of differentiation and the year of diagnosis.

## Results

**Frequencies of gynecological cancers:** during the study period, 682 cancers were diagnosed among which 342 were gynecological and breast cancers, leading to an overall frequency of 50.1%. The annual incidence of gynecological cancers was 34.2 cases on average (342/10).

**Figure 3:**
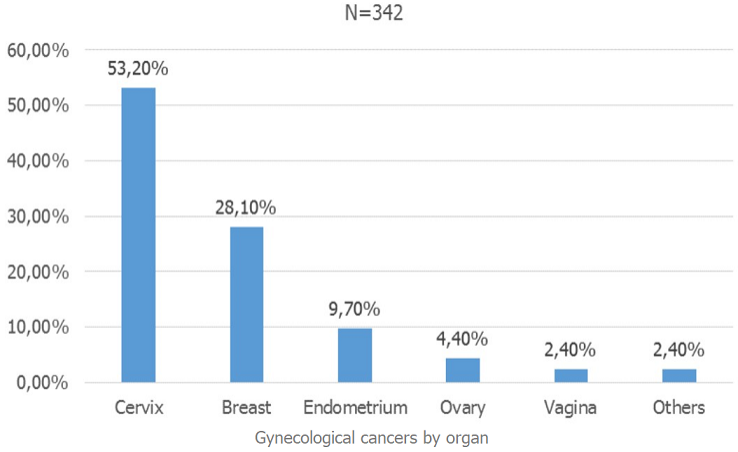
distribution of gynecological cancers by organ

**Distribution of gynecological cancers according to the affected organ:** Figure 3 shows distribution of gynecological cancers according to the affected organ. Cervical cancer was the most common with 53.2% (182 cases), followed by breast cancer with 28.1% (96 cases).

**Distribution of gynecological cancers by year of diagnosis:** distribution of gynecological cancers according to the year of diagnosis is shown in Table 1. The frequency of diagnosed gynecological cancers tended to increase from one year to another. The highest frequency was observed in 2016 with 23.7% (72) of cases, and the lowest frequency was observed in 2008 with 4.1% (14) cases.

**Table 1:** distribution of gynecological cancers by affected organ and year of diagnosis

	Affected organs										Total	
	Cervix		Breast		Endometrium		Ovary		Others			
Year of dg.	n=182		n=96		n=33		n=15		n=16		N=342	
	n	%	n	%	n	%	n	%	N	%	n	%
2008	07	3.8	02	2.1	03	9.1	02	13.3	00	00	14	4.1
2009	11	6.0	07	7.3	01	3.0	00	00	03	18.7	22	6.4
2010	08	4.4	10	9.4	02	6.1	01	6.7	00	00	21	5.3
2011	12	6.6	09	7.3	03	9.1	05	26.7	02	12.5	31	8.2
2012	17	9.3	10	9.4	05	15.2	05	33.3	01	6.3	38	10.0
2013	21	11.5	04	8.2	04	12.1	02	13.3	02	12.5	33	13.7
2014	14	7.8	05	3.1	03	9.1	00	00	02	12.5	24	7.0
2015	29	15.9	10	11.5	04	12.1	00	00	03	18.7	46	14.3
2016	39	21.5	26	29.2	05	15.2	00	00	02	12.5	72	23.7
2017	24	13.2	13	12.5	03	9.1	00	6.7	01	6.3	41	7.3

(dg.: diagnostis)

**Distribution of patients by age group and affected organ:** the distribution of patients by age group and affected organ is shown in Table 2. Cancers were more common in those who were 40 years old and above.

**Table 2:** distribution of patients by age group and affected organ (N=342)

	Affected organs										Total	
	Cervix		Breast		Endometrium		Ovary		Others			
Age (class)	N=182		N=96		N=33		N=15		N=16		N=342	
	n	%	n	%	N	%	N	%	N	%	n	%
[20-30[	05	2.8	04	4.2	04	12.1	02	13.3	00	00	15	4.4
[30-40[	27	14.8	14	14.6	01	3.0	01	6.8	04	25	47	13.7
[40-50[	54	**29.7**	30	**31.3**	02	6.1	02	13.3	04	25	92	26.9
[50-60[	34	18.6	24	25.0	14	**42.4**	05	33.3	04	25.0	81	23.7
[60-70[	31	17.0	14	14.5	06	18.2	05	33.3	01	6.3	57	16.7
[70-80[	30	16.5	10	10.4	05	15.2	00	00	03	18.7	48	14.0
[80-90[	01	0.6	00	00	01	3.0	00	00	00	00	02	0.6

**Distribution of patients by other socio-demographic characteristics:** Table 3 shows that more than half of the patients were married (56.7%), housewives (60.4%) and Christian (64.9%) while less than half of the patients 51 (45.95%) resided in Yaoundé.

**Table 3:** distribution by other socio-semographic characteristics

	Characteristics	Number (N=111)	Percentage (%)
Marital status	Married	63	56.7
	Widow	30	27.1
	Single	17	15.3
	Divorcee	01	0.9
Profession	No occupation	67	60.4
	Official	10	9.0
	shopping	11	9.9
	farmer	12	10.8
	Other	11	9.9
Religion	Catholic	49	44.1
	protestant	23	20.8
	Muslim	08	7.3
	Other	31	27.9
Residence	Yaounde	51	45.9
	Others	44	39.6
	NA	16	14.5

(NA: Non Available)

**Cervical cancer:** the histological types, aggression and degree of differentiation of cervical cancers were presented in Table 4. Squamous cell carcinoma was the most common histological type accounting for 86.8% (158). Invasive cervical cancers were the most common in 80.9% of cases. Among invasive cancers, 45.5% were well differentiated and 37.1% were moderately differentiated.

**Table 4:** histopathological Charateristics of cervical cancer

	Characteristics	Number (n)	Percentage (%)
Histological type (N = 182)			
	Squamous cell carcinoma	**158**	**86.8**
	Adenocarcinoma	21	11.7
	Adenosquamous carcinoma	01	0.5
	Mesonephrotic adenocarcinoma	01	0.5
	Angiosarcoma	01	0.5
Aggressiveness (N = 173)			
	Invasive	**140**	**80.9**
	Microinvasive	20	11.6
	In situ	13	7.5
Degree of differentiation (N = 132)			
	Well differentiated	**60**	**45.5**
	Moderately differentiated	49	37.1
	Little differentiated	23	17.4

**Breast cancer:** the histological types, aggressiveness and grades of Scarff, Bloom and Richardson breast cancers were grouped together and are presented in Table 5. The ductal and lobular carcinomas were the most common with 78.1% (75/96) and 16.7% (16/96) respectively. The majority, 92%, were invasive cancers. Grade II of Scarff, Bloom and Richardson was the most common representing 50.6% (39) while Grades I and III accounted for 16.9% (13) and 32.5% (25) of cases, respectively of our series.

**Table 5:** histopathological characteristics of breast cancer

	Characteristics	Number (n)	Percentage (%)
Histological type (N = 96)			
	Ductal carcinoma	75	78,1
	Lobular carcinoma	16	16,7
	Medullary carcinoma	02	2,1
	Colloid carcinoma	02	2,1
	Adenoid cystic carcinoma	01	1,0
Aggressiveness (N = 87)			
	Invasive	80	92,0
	In situ	07	8,0
Grade SBR (N=77)			
	SBR I	13	16,9
	SBR II	39	50,6
	SBR III	25	32,5

(SBR: Scarff Bloom Richardson )

**Cancer of the uterine body:** the histological types of cancers of the uterine body were distributed as shown in Table 6. Adenocarcinoma was the most recovered histological type with a proportion of 72.2% (26/36); followed by choriocarcinoma 13.8% (5/36).

**Table 6:** distribution according to the histological types of cancer of the body of the uterus

Histological type	Number (N=36)	Percentage (%)
Adenocarcinoma	26	72.2
Choriocarcinoma	05	13.8
Clear cell carcinoma	02	5.6
Carcinoma	01	2.8
Leiomyosarcoma	01	2.8
Stromal endometrial sarcoma	01	2.8

**Ovarian cancer:** the histological types of ovarian cancers are shown in Table 7. Serious cystadenocarcinoma was the most common histological type accounting for 46.7% (7/15), followed by mucinous cystadenocarcinoma 26.7% (4/15).

**Table 7:** distribution by histological type of ovarian cancer

Histological type	Number (N=15)	Percentage (%)
Serious cystadenocarcinoma	07	46.7
Mucinous cystadenocarcinoma	04	26.7
Clear cell adenocarcinoma	02	13.3
Burkitt's lymphoma	02	13.3

**Other cancers:** eight cases of vaginal cancer were identified, of which 87.5% (7/8 cases) were squamous cell carcinoma and 12.5% (1/8) were embryonic rhabdomyosarcoma. All vulvar cancers (05 cases) were squamous cell carcinomas and all trunk cancers were adenocarcinomas.

## Discussion

### Epidemiological characteristics of gynecological cancers

Average annual incidence of gynecological cancers in our series was 34.2 cases on average (342/10), which is lower than the 94.2 cases per year found by Sando *et al.* in 2014 at the Gyneco-obstetrics and Pediatric Hospital of Yaoundé [4]. This difference can be explained by the fact that, Gyneco-obstetrics and Pediatric Hospital of Yaoundé is the national reference hospital in oncological gynecology, meaning that it has a greater attendance to which can be added the proximity between the latter and the Yaoundé General Hospital, which houses the Yaoundé Medical Oncology Reference Center. The distribution of gynecological cancers according to the year of diagnosis shows that the number of cancers diagnosed per a year tended to increase over time, with a peak frequency in 2016 for cervical cancers, ie 21.4% (39/182) and breast 27.1% (26/96). These results can be explained by the existence of a breast and cervical cancer screening unit at the University Teaching Hospital of Yaoundé since 2009 and the organization of a cervical and breast cancer screening campaign in 2016. In addition, it is likely that doctors increasing prescription of screening and histological examinations over this time period. The unavailability of trained personnel needed to perform this service (two gynecologists and a nurse); could explain the decrease in frequency observed in 2014.

Regarding the distribution of gynecological cancers according to the affected organ, cervical cancer was the leading gynecological cancer accounting for 53.2% of cases, this predominance of cervical cancer was found by several authors, including Tebeu *et al.* in 2009, which found 57% in Maroua and Sando *et al.* in 2014, who found 49.5% at University Teaching Hospital of Yaoundé. However, this proportion is lower than that found by Engbang *et al.* in 2015 in the Littoral and N'Dah *et al.* in 2014 in Côte d'Ivoire, which reached 72.3% and 82.8% respectively [2, 5-7]. This difference could be explained by the fact that the latter authors did not include breast cancer in their series. This high frequency of cervical cancer in our context can be explained by its multiple risk factors including: factors that increase the risk of infection with human papillomavirus (HPV), such as unprotected premature intercourse (under 17 years of age) and multiple sexual partners, multiparity, oral contraceptives and immunodeficiency [8]. It can also be justified by the absence of vaccination against HPV and a cervical cancer screening policy in our context. Indeed, vaccination against HPV serotypes 16 and 18, which are found in 70% of cases of cervical cancer, has been available for of young girls (9-14 years old) in Cameroon since 2014 [1]. Breast cancer was the second most common type of cancer in our series with a proportion of 28.1%, which is similar to that of Sando *et al.* in 2014 at the Gyneco-Obstetrics and Pediatric Hospital Yaoundé and differs from that of Orock *et al.* in 2012. In contrast, a study using the cancer registry found that breast cancer is the leading cancer in women [4, 9]. These discordant results raise the issue that, the cancer registry in Cameroon is not fully functional. However, breast cancer is the most prevalent of women's cancers in the world in both developed and developing countries. It accounts for 51% of women's cancers in Burkina Faso, 75.3% in Madagascar, 40.4% of gynecological cancers in Niger [10-12].

Endometrial cancer was third most common cancer in our series, accounting for 9.7%; this result is similar to that of Engbang *et al.* who found 11.6% in 2015 in the Littoral region of Cameroon and Agboeze *et al.* found a proportion of 10.1% in Nigeria in 2015 [6, 13]. This proportion, however, is lower than those found in developed countries, especially the 40.1% reported in Japan by Yamagami *et al.* in 2017 and 45.9% in Turkey according to Gultekin *et al.* in 2017 [14, 15]. This difference could be explained by the fact that, unlike cervical cancer, where screening methods are known and screening programs are operational and effective in these countries, there is no method of screening for endometrial cancer. For cervical cancer, the mean age was 52.8±13.5 years with extremes of 26 and 82 years; this result was similar to those of Sando *et al.* in 2014, who had an average age of 52.4±3.8 years for cervical cancer in Yaoundé and Engbang *et al.* who found 51.2±11.9 years in the Littoral [4, 6]. This result could be explained by the 10-20 year long evolution of the pre-cancerous lesion to invasive cancer and by the lack of a cancer screening program in general in our context. Fewer than half of the patients resided in Yaoundé and more than half were admitted to the University Teaching Hospital Yaoundé by reference. These results can be explained by the reduced number of diagnostic structures across the national territory which are concentrated in the larger metropolitan areas, therefore if there is a suspicion of cancer in a first-level health facility, patients will be conducted to a referral hospital, including the University Teaching Hospital Yaoundé.

### Histopathological features of gynecological cancers

**Cervical cancer**: squamous cell carcinoma was the predominant histological type accounting for 86.8% (158 of 182 cases) followed by adenocarcinoma. This result is consistent with the literature where this proportion varies between 72-94% [2, 4, 6, 10, 14-16].

**Breast cancer**: cancer: 78.1% (75 of 96) of breast cancers were ductal carcinomas. This result is consistent with the literature, where this proportion varies between 64.9-92.5% [4, 17, 18]. In addition, according to the histopronostic grade, breast cancers were predominantly of intermediate prognosis (grade II), followed by those with a poor prognosis (grade III); this result is similar to those found by Engbang *et al.* in Cameroon and Belhafiane *et al.* in Morocco [19, 20].

**Endometrial cancer**: adenocarcinoma was the most common histologic type of endometrial cancer accounting for 72.2% (26/36) of cases. This result is comparable to those found by Sando *et al.* at Gyneco-Obstetrics and Pediatric Hospital of Yaoundé (90.50%), N'Dah *et al.* in Côte d'Ivoire 67,7% and 86.6% by Shahid *et al.* in Pakistan [2, 4, 21]. These results are explained by the fact that the lining of the endometrium is covered by a simple cylindrical epithelium.

**Ovarian cancer**: epithelial cancers were the most common type of ovarian cancer with 86.7%, with serous cystadenocarcinoma being the most common. This predominance of epithelial cancers has been found in the literature in proportions varying between 68-93% [2, 4, 6, 22, 23]. The predominant histologic type of cancers of the vagina and vulva was squamous cell carcinoma in proportions of 88.9% (8 out of 9) and 100% (3 out of 3), respectively, which corroborates with data from the literature [2, 6]. This result is explained by the fact that same type of epithelium, namely the pluristratified squamous epithelium, covers the mucous membranes of the vagina and vulva.

## Conclusion

In this study, we aimed to describe the epidemiological and clinical characteristics of gynecological cancers at the University Teaching Hospital of Yaoundé. Our results indicate that gynecological cancers represent 50.1% of women's cancers and that the most common are cancers of the cervix and breast. Most of the gynecological cancer patients were housewives, multiparous and above 50 years of age. The most common histopathological types were squamous cell carcinoma for cervical cancer and ductal carcinoma for breast cancer. Based on these findings, sensitization should be strengthened and the routine prescription of cervical and breast cancer screening tests should begin at 30 and 40 years of age respectively.

### What is known about this topic

- Cancer is the third leading cause of death in developing countries;
- Gynecological cancers account for 54.3% of women's cancers in Africa;
- There are several histopathological types of gynecological cancers.

### What this study adds

- Calculation of the global and specific frequencies of gynecological and mammary cancers;
- Description of socio-demographic characteristics of gynecological cancer patients;
- Determination of the most frequent histopathological types of gynecological cancers.

## Competing interests

The authors declare no competing interests.
